# Increasing the Analytical Sensitivity by Oligonucleotides Modified with *Para*- and *Ortho*-Twisted Intercalating Nucleic Acids – TINA

**DOI:** 10.1371/journal.pone.0020565

**Published:** 2011-06-03

**Authors:** Uffe V. Schneider, Imrich Géci, Nina Jøhnk, Nikolaj D. Mikkelsen, Erik B. Pedersen, Gorm Lisby

**Affiliations:** 1 QuantiBact Inc, Hvidovre, Denmark; 2 Nucleic Acid Center and Department of Chemistry, University of Southern Denmark, Odense, Denmark; University of Pennsylvania, United States of America

## Abstract

The sensitivity and specificity of clinical diagnostic assays using DNA hybridization techniques are limited by the dissociation of double-stranded DNA (dsDNA) antiparallel duplex helices. This situation can be improved by addition of DNA stabilizing molecules such as nucleic acid intercalators. Here, we report the synthesis of a novel *ortho*-Twisted Intercalating Nucleic Acid (TINA) amidite utilizing the phosphoramidite approach, and examine the stabilizing effect of *ortho*- and *para*-TINA molecules in antiparallel DNA duplex formation. In a thermal stability assay, *ortho*- and *para*-TINA molecules increased the melting point (Tm) of Watson-Crick based antiparallel DNA duplexes. The increase in Tm was greatest when the intercalators were placed at the 5′ and 3′ termini (preferable) or, if placed internally, for each half or whole helix turn. Terminally positioned TINA molecules improved analytical sensitivity in a DNA hybridization capture assay targeting the *Escherichia coli rrs* gene. The corresponding sequence from the *Pseudomonas aeruginosa rrs* gene was used as cross-reactivity control. At 150 mM ionic strength, analytical sensitivity was improved 27-fold by addition of *ortho*-TINA molecules and 7-fold by addition of *para*-TINA molecules (versus the unmodified DNA oligonucleotide), with a 4-fold increase retained at 1 M ionic strength. Both intercalators sustained the discrimination of mismatches in the dsDNA (indicated by ΔTm), unless placed directly adjacent to the mismatch – in which case they partly concealed ΔTm (most pronounced for *para*-TINA molecules). We anticipate that the presented rules for placement of TINA molecules will be broadly applicable in hybridization capture assays and target amplification systems.

## Introduction

The stability of double-stranded DNA (dsDNA) is naturally limited to allow cellular processes that require helix dissociation such as gene transcription, gene regulation and cell division. However, the sensitivity of DNA diagnostic assays depends upon the stability of dsDNA helices. The analytical sensitivity of an assay can be improved by decreasing stringency, but at the risk of cross-reactivity to other targets.

**Figure 1:**
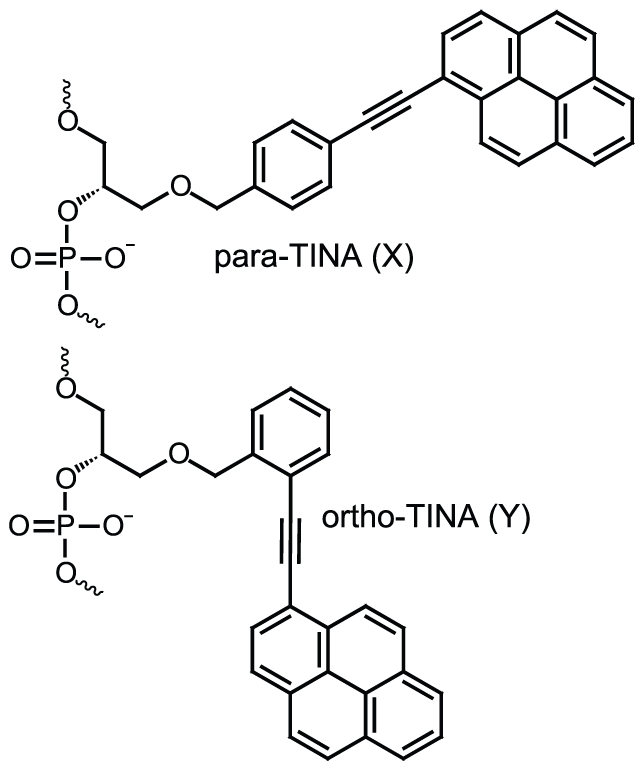
Structure of Twisted Intercalating Nucleic Acids showing the *para*-TINA (X) and *ortho*-TINA (Y) molecules.

To increase the stability of dsDNA, a number of DNA stabilizing molecules have been developed [1]–[13]. DNA stabilizing molecules comprising intercalators, except TINA and AMANY were developed to increase the stability of Watson-Crick based antiparallel duplex formation[8], [10]–[12], [14], [15]. TINA and AMANY molecules were designed to stabilize Hoogsteen based triplex and parallel duplex formation[14], [15]. Surprisingly, a recent thermal stability study of TINA molecule design demonstrated that changing the attachment of the ethynylpyrene functional group from *para* to *ortho* (Figure 1) produced significant changes in antiparallel duplex stability [12], [16]. Internal insertion of *ortho*-TINA ((*R*)-1-*O*-[2-(1-pyrenylethynyl)phenylmethyl]glycerol) molecule stabilized the Watson-Crick based antiparallel duplex formation, in contrast to the original *para*-TINA ((*R*)-1-*O*-[4-(1-pyrenylethynyl)phenylmethyl]glycerol) molecule which was only shown to stabilize antiparallel DNA duplex formation when placed at the 5′ terminal [14]. Here, we investigate the optimal placement of *para*- and *ortho*-TINA molecules in antiparallel DNA duplex formation, and report the first evaluation of *para*- and *ortho*-TINA molecules in antiparallel DNA duplex based hybridization capture assays.

In addition, we report the synthesis of a novel *ortho*-TINA amidite using the phosphoramidite approach. Until now, *ortho*-TINA containing oligonucleotides has been synthesized by postsynthetic oligonucleotide modification using the Sonogashira Pd-catalyzed coupling reaction [12], [16]. Although this approach is advantageous for the screening of different intercalators [14], to achieve sufficiently high coupling yields the reaction has to be repeated several times with fresh portions of the Sonogashira mixture. This can affect the subsequent oligonucleotide purification process. The phosphoramidite approach permits the production of a large number of oligonucleotides with several *ortho-*TINA molecule insertions in the sequences.

We find that inclusion of *para-* as well as *ortho*-TINA molecules in an oligonucleotide is capable of improving the analytical sensitivity of probe hybridization without increasing cross-reactivity in a competitive antiparallel duplex hybrization capture assay. We anticipate that TINA molecules will enable a general improvement in the performance of future clinical diagnostic assays based upon conventional hybridization, as well as Polymerase Chain Reaction (PCR) and other primer-based enzymatic target amplification systems.

## Results

### Synthesis of ortho-TINA amidite

As an alternative to the traditional method of postsynthetic oligonucleotide modification, we prepared the *ortho-*TINA monomer for use on a DNA synthesis platform via the more convenient phosphoramidite approach. The newly designed *ortho-*TINA phosphoramidite was synthesized in two steps from a known starting compound [12].

**Figure 2:**
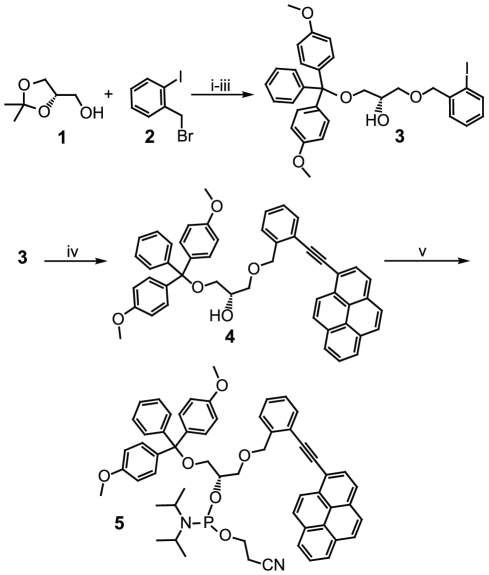
Synthesis of *ortho*-TINA phosphoramidite. i–iii = synthesis of starting compound; iv–v = two-step synthesis of *ortho*-TINA phosphoramidite [Reagents and conditions: i) KOH, toluene, reflux; ii) 80% aq. CH_3_COOH, room temperature (RT); iii) DMTCl, pyridine, RT; iv) Et_3_N, N_2_, Pd(PPh_3_)_2_Cl_2_, CuI, 1-Ethynylpyrene, RT; v) NC(CH_2_)_2_OP(N*^i^*Pr_2_)_2_, diisopropylammonium tetrazolide, CH_2_Cl_2_, 0°C to RT.] **1** = *S*-(+)-2,2-dimethyl-1,3-dioxalane-4-methanol; **2** = 2-iodobenzylbromide; **3** =  (S)-1-O-(4,4′-dimethoxytriphenylmethyloxy)-3-O-(2-iodobenzyloxy)propan-2-ol; **4** = DMT-protected *ortho*-TINA; **5** = *ortho*-TINA phosphoramidite.

Full details of the synthesis procedure are provided in the materials and methods section, and in Supplementary Data S1. In brief (Figure 2), the starting compound (**3**) was prepared (80% overall yield) in three steps from commercially available compounds *S*-(+)−2,2-dimethyl-1,3-dioxalane-4-methanol (**1**) and 2-iodobenzylbromide (**2**). In the first step of the *ortho-*TINA phosphoramidite synthesis, 1-ethynylpyrene was coupled to compound **3** using the Sonogashira coupling mixture [14]. To eliminate oxygen, the reaction mixture was degassed with nitrogen prior to the addition of tritylated compound **3**; when the reaction mixture was not degassed, the product yield decreased significantly. DMT-protected *ortho-*TINA (**4**) was obtained as yellow foam (85% yield), and its structure was confirmed by NMR spectrometry. Finally, the secondary hydroxyl group was phosphatized. Signals in the ^31^P NMR spectrum with chemical shifts of 148.9 and 149.3 ppm, respectively, confirmed the formation of the phosphoramidite (**5**).

### Thermal stability of *para*- and *ortho*-TINA modified oligonucleotides

To determine the optimal placement of *para*- and *ortho*-TINA molecules for stabilizing antiparallel DNA duplexes, we used a fluorescence resonance energy transfer (FRET) based high-speed melting curve method, as described and validated previously [17], [18]. An 18-mer oligonucleotide from the *Escherichia coli* (*E. coli*) *rrs* gene (base pair 772–789) was used as the target. Table 1 shows the melting points (Tm) of *para*- and *ortho*-TINA modified oligonucleotides and changes in Tm associated with mismatches in the target strand (ΔTm). The full data set can be found in Table S1. *Ortho*- and *para*-TINA molecule insertions in the oligonucleotide increased Tm when placed terminally on the oligonucleotide, although the *para*-TINA molecule produced the greater increase. Maximum stability was reached when there was a modification at both termini. Placed internally, *para*-TINA molecules decreased Tm in all positions, especially when at the center of the oligonucleotide, whereas the positive effect of *ortho*-TINA molecules on Tm was neutralized towards the center of the oligonucleotide. The combination of a terminal *para-* or *ortho*-TINA molecule with an internal *para-* or *ortho*-TINA molecule showed the highest increases in Tm when the two modifications were separated by six or twelve nucleotides, equaling a half or complete helix turn. Both *ortho*-TINA and (especially) *para*-TINA molecules were found to partly conceal the ΔTm of a mismatch immediately next to them, but when the mismatch was moved one or more nucleotides away, they had no effect on ΔTm. The stabilizing effect of *para*- and *ortho*-TINA molecules increased when the oligonucleotide sequence was shortened from eighteen to sixteen nucleotides.

**Table 1:** Melting point evaluation of TINA modified oligonucleotides.

Antiparallel duplex sequences with/without *para*- and *ortho*-TINA		Melting point, Tm (°C)					
		*E. coli rrs* target sequence: match 5′ TGGGGAGCAAACAGGATT-ATTO495 3′ (D-624)	Effect of TINA on Tm	*E. coli rrs* target sequence: mismatch 5′ TGGGGAGCATACAGGATT-ATTO495 3′ (D-634)	Mismatch ΔTm	*E. coli rrs* target sequence: mismatch 5′ TGGGGAGCAATCAGGATT-ATTO495 3′ (D-809)	Mismatch ΔTm
**Unmodified sequence (no TINA)**							
LC D-643	ATTO590- AAT CCT GTT TGC TCC CCA	68.9		61.6	−7.3	61.1	−7.8
**Modification with ** ***ortho*** **-TINA(Y)**							
LC D-644	ATTO590-**Y**AAT CCT GTT TGC TCC CCA	72.8	3.9	65.0	−7.8	64.9	−7.9
LC D-645	ATTO590- AAT**Y**CCT GTT TGC TCC CCA	69.5	0.6	60.9	−8.6	60.2	−9.3
LC D-646	ATTO590- AAT CCT**Y**GTT TGC TCC CCA	69.2	0.3	61.9	−7.3	61.1	−8.1
LC D-647	ATTO590- AAT CCT GTT**Y**TGC TCC CCA	68.8	−0.1	64.8	−4.0*	61.6	−7.2
LC D-648	ATTO590- AAT CCT GTT TGC**Y**TCC CCA	69.8	0.9	62.0	−7.8	61.4	−8.4
LC D-649	ATTO590- AAT CCT GTT TGC TCC**Y**CCA	72.0	3.1	64.1	−7.9	63.8	−8.2
LC D-650	ATTO590- AAT CCT GTT TGC TCC CCA**Y**	70.2	1.3	62.2	−8.0	62.3	−7.9
LC D-653	ATTO590-**Y**AAT CCT GTT**Y**TGC TCC CCA	72.8	3.9	68.7	−4.1*	65.6	−7.2
LC D-655	ATTO590-**Y**AAT CCT GTT TGC TCC CCA**Y**	74.6	5.7	66.5	−8.1	66.1	−8.5
LC D-660	ATTO590-**Y**AAT CCT GTT**Y**TGC TCC CCA**Y**	72.8	3.9	68.4	−4.4*	65.6	−7.2
**Modification with ** ***para*** **-TINA(X**)							
LC D-665	ATTO590-**X**AAT CCT GTT TGC TCC CCA	73.7	4.8	66.3	−7.4	65.6	−8.1
LC D-666	ATTO590- AAT**X**CCT GTT TGC TCC CCA	67.9	−1.0	58.7	−9.2	58.1	−9.8
LC D-667	ATTO590- AAT CCT**X**GTT TGC TCC CCA	65.2	−3.7	57.9	−7.3	57.8	−7.4
LC D-668	ATTO590- AAT CCT GTT**X**TGC TCC CCA	63.1	−5.8	59.6	−3.5*	57.4	−5.7
LC D-669	ATTO590- AAT CCT GTT TGC**X**TCC CCA	64.2	−4.7	55.6	−8.6	55.0	−9.2
LC D-670	ATTO590- AAT CCT GTT TGC TCC**X**CCA	67.7	−1.2	58.9	−8.8	58.8	−8.9
LC D-671	ATTO590- AAT CCT GTT TGC TCC CCA**X**	67.7	−1.2	60.8	−6.9	60.4	−7.3
LC D-674	ATTO590-**X**AAT CCT GTT**X**TGC TCC CCA	69.6	0.7	65.8	−3.8*	63.8	−5.8
LC D-679	ATTO590- AAT CCT GTT**X**TGC TCC CCA**X**	64.4	−4.5	60.6	−3.8*	57.8	−6.6
LC D-676	ATTO590-**X**AAT CCT GTT TGC TCC CCA**X**	76.8	7.9	68.8	−8.0	68.5	−8.3
LC D-681	ATTO590-**X**AAT CCT GTT**X**TGC TCC CCA**X**	69.5	0.6	65.7	−3.8*	63.9	−5.6
**Modification with ** ***para*** **(X)- and ** ***ortho*** **(Y)-TINA**							
LC D-748	ATTO590-**X**AAT**Y**CCT GTT TGC TCC CCA	73.2	4.3	65.3	−7.9	65.0	−8.2
LC D-749	ATTO590-**X**AAT CCT**Y**GTT TGC TCC CCA	74.6	5.7	67.6	−7.0	66.3	−8.3
LC D-750	ATTO590-**X**AAT CCT GTT**Y**TGC TCC CCA	73.7	4.8	69.9	−3.8*	67.3	−6.4
LC D-751	ATTO590-**X**AAT CCT GTT TGC**Y**TCC CCA	74.8	5.9	67.4	−7.4	66.7	−8.1
LC D-754	ATTO590- AAT CCT GTT**Y**TGC TCC CCA**X**	69.8	0.9	64.9	−4.9*	61.6	−8.2
LC D-756	ATTO590-**X**AAT CCT GTTYTGC TCC CCA**X**	73.9	5.0	70.0	−3.9*	67.7	−6.2
**Shortened sequence with ** ***para*** **(X)- or ** ***ortho*** **(Y)-TINA**							
LC D-661	ATTO590- AAT CCT GTT TGC TCC C	66.0		58.0	−8.0	57.7	−8.3
LC D-662	ATTO590-**Y**AAT CCT GTT TGC TCC C	70.3	4.3	61.9	−8.4	61.5	−8.8
LC D-663	ATTO590- AAT CCT GTT TGC TCC C**Y**	69.0	3.0	60.5	−8.5	60.4	−8.6
LC D-664	ATTO590-**Y**AAT CCT GTT TGC TCC C**Y**	73.9	7.9	65.4	−8.5	65.1	−8.8
LC D-682	ATTO590-**X**AAT CCT GTT TGC TCC C	71.1	5.1	63.2	−7.9	62.9	−8.2
LC D-683	ATTO590- AAT CCT GTT TGC TCC C**X**	68.6	2.6	60.1	−8.5	59.8	−8.8
LC D-684	ATTO590-**X**AAT CCT GTT TGC TCC C**X**	75.6	9.6	66.5	−9.1	66.3	−9.3

Change in Tm and ΔTm of Watson-Crick based antiparallel duplexes stabilized by *para*-TINA (X) and/or *ortho*-TINA (Y) molecules. Tm was determined using 0.5 µM of each strand in 50 mM phosphate buffer, pH 7.0, with 100 mM NaCl and 0.1 mM EDTA. Tm was defined as the peak of the first derivate using both annealing and dissociation curves. Base mismatches are underlined and marked in bold black. *Mismatch adjacent to TINA.

### Effect of ionic conditions on dsDNA *E. coli rrs* gene PCR product capture by *para*- and *ortho*-TINA containing oligonucleotides

**Figure 3:**
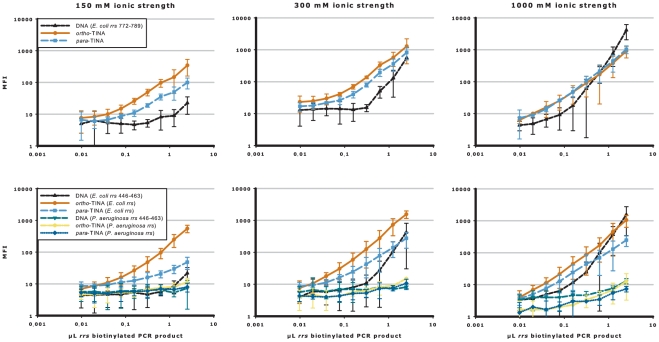
TINA modified oligonucleotides increase the analytical sensitivity in buffer of increasing ionic strength. Competitive annealing of *ortho*- or *para*-TINA terminally modified oligonucleotides, compared with unmodified DNA oligonucleotide, to denatured PCR products in buffer of increasing ionic strength. *E. coli rrs* biotinylated PCR product was captured by unmodified DNA oligonucleotide (▴) and *ortho*-TINA (•) or *para*-TINA (▪) modified oligonucleotides targeting *E. coli rrs* base pairs 772–789 and 446–463, with unmodified DNA oligonucleotide (▾), *ortho-*TINA (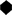) or *para*-TINA (⧫) modified oligonucleotides targeting *P. aeruginosa* base pairs 446–463 as cross-reactivity control. In experiments targeting base pairs 446–463, a conventional DNA helper oligonucleotide (base pair target 464–483) was included. Experiments were performed in phosphate buffer, pH 7.0, with 0.03% Triton X-100 and increasing ionic strength (100–1,000 mM) at 52°C. Data are presented as mean raw MFI with 95% confidence intervals. Full data are shown in Figure S1.

Until now, the effects of TINA molecules have only been evaluated by Tm analyses, which are good model systems, but do not provide information on how TINA-modified oligonucleotides will perform as competitive annealing probes. To address this issue, we used the Luminex® 200™ instrument to analyze the capture of denatured biotinylated *E. coli rrs* PCR product by magnetic microspheres coated with oligonucleotide sequences targeting base pairs 772–789 from the *E. coli rrs* gene. Figure 3 and Figure S1 show the capture of biotinylated *rrs* PCR product (in two-fold dilution series from 2.5 µL to 0.0098 µL *rrs* PCR product) by unmodified DNA oligonucleotides and oligonucleotides terminally modified with *para*- or *ortho*-TINA molecules in buffers of increasing ionic strength (100–1,000 mM monovalent cation). The overall level of median fluorescence intensity (MFI) was generally higher at greater ionic strength. In 150 mM buffer, the *ortho*-TINA modified oligonucleotide increased the analytical sensitivity 27-fold and the *para*-TINA modified oligonucleotide increased the analytical sensitivity seven-fold, compared with the unmodified DNA oligonucleotide. In 300 mM buffer, *ortho*-TINA modified oligonucleotide increased analytical sensitivity eleven-fold and *para*-TINA modified oligonucleotide six-fold, and even at 1,000 mM, a four-fold increase in analytical sensitivity was observed with both modified oligonucleotides compared with the unmodified equivalent.

To ensure that the increased analytical sensitivity was target sequence independent, the capture sequence was changed to base pairs 446–463 of the *E. coli rrs* gene. The corresponding sequence from *Pseudomonas aeruginosa* (*P. aeruginosa*) *rrs* gene is the most closely related sequence among the human pathogens. Consequently, *P. aerugionsa* was used as cross-reactivity control and contains a cluster of four mismatches to the *E. coli* sequence. A helper oligonucleotide (targeting *E. coli rrs* gene base pairs 464–483) was also added to prevent secondary structure formation (not required for base pairs 772–789). Changing the target sequence did not change the capture curves for the unmodified DNA and *ortho*-TINA modified oligonucleotides, whereas *para*-TINA modified oligonucleotides did not perform as well as for the 772–789 base pair target. There was no cross-reactivity with the *P. aeruginosa* control sequence.

### Effect of hybridization temperature on dsDNA *E. coli rrs* gene PCR product capture by *para*- and *ortho*-TINA containing oligonucleotides

**Figure 4:**
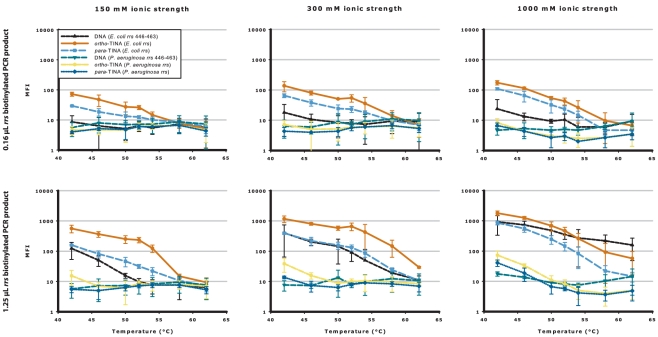
TINA modified oligonucleotides increase the analytical sensitivity over a wide range of annealing temperatures. Competitive annealing of *ortho*- or *para*-TINA terminally modified oligonucleotides, compared with unmodified DNA oligonucleotide, to denatured PCR products at annealing temperatures from 42°C to 62°C. *E. coli rrs* biotinylated PCR product (at 0.16 µL and 1.25 µL concentrations) was captured by unmodified DNA oligonucleotide (▴) and *ortho*-TINA (•) or *para*-TINA (▪) modified oligonucleotides targeting *E. coli rrs* base pairs 446–463, with unmodified DNA oligonucleotide (▾), *ortho-*TINA (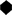) or *para*-TINA (⧫) modified oligonucleotides targeting *P. aeruginosa* base pairs 446–463 as cross-reactivity control. In all experiments, a conventional DNA helper oligonucleotide targeting *E. coli rrs* base pairs 464–483 was included. Experiments were performed in phosphate buffer, pH 7.0 with 0.03% Triton X-100, and 150 mM, 300 mM and 1000 mM ionic strengths at temperatures from 42°C to 62°C. Data are presented as mean raw MFI with 95% confidence intervals.

To investigate whether the modulating effect of TINA molecules was temperature specific, the DNA hybridization assay was repeated at annealing temperatures from 42–62°C at three different ionic strengths and with two different concentrations for the *E. coli rrs* gene 446–463 base pair target sequence. *P. aeruginosa* was used as a cross-reactivity control sequence. As shown in Figure 4, the relative MFI of the terminally modified *ortho*- and *para*-TINA and unmodified DNA oligonucleotides remained unchanged between 42°C and 52°C (temperature used in the ionic experiments), with the modified oligonucleotides generally providing the highest MFI. Above 52°C the difference in MFI rapidly diminished due to loss of signal. As expected, the level of cross-reactivity with the *P. aeruginosa* oligonucleotides rose with increasing ionic strength as the annealing temperatures decreased.

### Effect of unlabeled helper oligonucleotide on dsDNA *E. coli* rrs gene PCR product capture

Previous studies have shown that the analytical sensitivity of 16S *E. coli* rRNA nucleotide 446–463 capture can be improved by helper nucleotides that prevent the formation of secondary structures in the RNA [19]. This is in contrast to 16S *E. coli* rRNA nucleotide 772–789 for which no secondary structure has been found. Accordingly, in the studies reported here, we included an unlabeled DNA helper oligonucleotide targeting *E. coli rrs* gene base pairs 464–483 when capturing the *E. coli rrs* gene base pair 446–463 sequence, to avoid formation of secondary structures in the denatured single stranded DNA.

**Figure 5:**
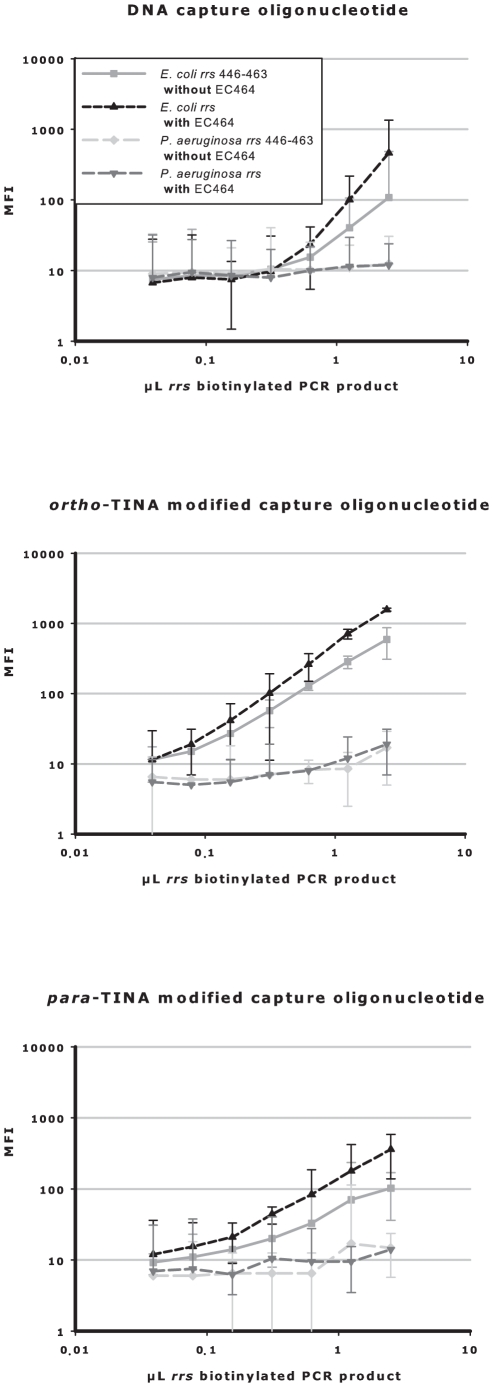
DNA helper oligonucleotides increase assay sensitivity independently of TINA modifications in the capture oligonucleotides. Competitive annealing of unmodified DNA and *ortho*- or *para*-TINA terminally modified oligonucleotides to denatured PCR products, with and without an unmodified DNA helper oligonucleotide. *E. coli rrs* biotinylated PCR product was captured by unmodified DNA oligonucleotide (top), or terminally modified *ortho*-TINA (middle) or *para*-TINA (bottom) oligonucleotides targeting *E. coli* or *P. aeruginosa rrs* base pairs 446–463, with and without an unmodified DNA helper oligonucleotide (EC464; targeting *E. coli rrs* base pairs 464–483). Experiments were performed in phosphate buffer, pH 7.0, with 0.03% Triton X-100 and 300 mM monovalent cations at 52°C. Data are presented as mean raw MFI with 95% confidence intervals.

As shown in Figure 5, we also examined the individual effect of this DNA helper oligonucleotide on analytical sensitivity when targeting *E. coli rrs* gene base pairs 446–463. Addition of the helper oligonucleotide increased the analytical sensitivity of the unmodified DNA and *ortho*- and *para*-TINA modified oligonucleotides by approximately two-fold. As shown in earlier experiments (Figure 3), targeting base pair 446–463 with TINA/DNA modified oligonucleotides plus the helper nucleotide (to relieve secondary structure), gave similar levels of capture sensitivity to those obtained when targeting base pair 772–789 (no secondary structure).

## Discussion

In the current paper, we have characterized the stabilizing effect and established design rules for placement of *ortho*- and *para*-TINA molecules into Watson-Crick based antiparallel DNA duplexes. According to thermal stability analyses, both *para*- and *ortho*-TINA molecules should be placed terminally in the nucleotide sequence, and preferably on both the 5′ and 3′ terminal positions to achieve a maximum increase in Tm. Placement of *para*-TINA molecules at the 5′ and 3′ termini gave the most pronounced increase in Tm compared to *ortho*-TINA molecules. The stabilizing effect of *para*- and *ortho*-TINA molecules changes when they are placed internally in the oligonucleotide sequence. *Ortho*-TINA molecules have either a positive effect or no effect on Tm, whereas *para*-TINA molecules decrease Tm when placed internally. However, neither *para*- nor *ortho*-TINA molecules interfere with mismatch-induced ΔTm, unless they are placed internally directly adjacent to the mismatch. Overall, when several TINA molecules are placed in an oligonucleotide, the highest increase in Tm is observed if they are placed at the 5′ and 3′ terminal positions (preferable) or, if placed internally as well, with the modifications separated by a half or whole helix turn.

The present thermal stability study was done using a single target sequence (the *E. coli rrs* gene base pair 772–789). The validity of the design rules are therefore still to be established, but the design rules suggested in this paper are in concordance with previously published thermal stability data on nucleic acid intercalator molecules in other target sequences [11]–[16], [20]. The design rules identified in this study are also identical to the design rules we established previously for placement of *para*-TINA molecules into Hoogsteen based parallel DNA triplex formations [18]. Since thermal stability data for a number of different nucleic acid intercalating molecules are in perfect agreement with the herein presented design rules [11]–[16], [20], we speculate whether these design rules might represent general design rules for placement of intercalator molecules into Watson-Crick based antiparallel duplex and Hoogsteen type triplex formations.

Previously, *para*-TINA has been tested for triplex and quadruplex hybridization in cellular systems [21], [22], but the present study is the first evaluation of *para*- and *ortho*-TINA molecules in antiparallel DNA duplex based hybridization capture assays. The pronounced increase in analytical sensitivity conferred by *para*- and *ortho*-TINA molecules in antiparallel DNA duplex hybridization is note-worthy, especially since the increased analytical sensitivity is seen for two different target sequences, with and without a helper oligonucleotide. In addition, the specificity of the signal is maintained without cross-hybridization under a wide range of ionic conditions (100 mM to 1 M monovalent cations).

As previously stated, the corresponding sequence from the *P. aeruginosa rrs* gene was used as a cross-reactivity control in the hybridization capture assay, as it is the most closely related sequence among the known human pathogens. This sequence contains a cluster of four mismatches to the *E. coli* sequence, so a closer related sequence would have been desirable from a pure “cross-reactivity control” point of view. However, we decided to use the *P. aeruginosa rrs* gene sequence as cross-reactivity control, since we wanted the capture of biotinylated PCR product in the hybridization capture assay to reflect the clinical diagnostics reality the most. So, the true impact of TINA molecules on oligonucleotide cross-reactivity is still to be established.

The *E. coli rrs* gene base pair 772–789 target sequence was used in both the thermal stability study as well as the antiparallel duplex based hybridization capture assay. In the thermal stability study, placement of *par*a-TINA molecules at the 5′ and 3′ termini gave the most pronounced increase in Tm compared to *ortho*-TINA molecules, but for capture of denatured *E. coli rrs* PCR product the analytical sensitivity was highest for *ortho*-TINA modified oligonucleotides. The thermal stability study reflects the temperature at which the fluorescence signal is changing at the highest rate, whereas the analytical sensitivity established in the hybridization capture assay reflects the hybridization to the target sequence in competitive annealing with the complementary strand of the PCR product. So even though the *para*-TINA modified oligonucleotides caused the highest Tm, the *ortho*-TINA modified oligonucleotides performed better in the competitive annealing hybridization capture assay.

Since addition of *ortho*-TINA in particular to the oligonucleotides increases the analytical sensitivity, we expect that *ortho*-TINA molecules, in particular, will be beneficial for increasing sensitivity, without compromising target specificity, in future clinical diagnostic assays, based on target hybridization capture as well as in target amplification systems. An example could be placement of an *ortho*-TINA molecule at the 5′ end of PCR primers to increase efficacy of primer annealing, and thereby the overall efficacy in quantitative as well as end-point PCR reactions.

## Materials and Methods

### Synthesis of *ortho*-TINA amidite

Solvents were dried prior to use. All chemicals were obtained from Sigma-Aldrich (Brøndby, Denmark) and were used as purchased. The silica gel (0.040–0.063 mm) used for column chromatography was purchased from Merck & Co Inc. (Whitehouse Station, NJ, USA). Solvents used for column chromatography were distilled prior to use.

NMR spectra were measured on a Varian Gemini 2000 spectrometer at 300 MHz for ^1^H using TMS (δ: 0.00) as an internal standard, at 75 MHz for ^13^C using CDCl_3_ (δ: 77.0) as an internal standard, and at 125.5 MHz using H_3_PO_4_ (δ: 77.0) as an internal standard. Mass spectra of the synthesized compounds were determined on the Ionspec 4.7 Tesla HiResMALDI Ultima Fourier transform (FT) mass spectrometer (Ion Spec, Irvine, CA, USA) controlled by the OMEGA data system.

Starting compound (**3**) was prepared according to the procedure of Filichev et al. [12] – refluxing *S*-(+)-2,2-dimethyl-1,3-dioxalane-4-methanol (**1**) with 2-iodobenzylbromide (**2**) in the presence of potassium hydroxide (i), followed by *in situ* deprotection of the intermediate with 80% acetic acid at room temperature (RT) (ii) (Figure 2). Subsequently, the primary hydroxyl group was selectively protected by dimethoxytrityl chloride under basic conditions (iii) to form tritylated compound **3**.

1-Ethynylpyrene coupling (iv) was accomplished using the Sonogashira coupling mixture [14]. The reaction mixture was degassed with nitrogen prior to addition of tritylated compound **3**. DMT-protected *ortho-*TINA (**4**) was obtained as yellow foam, and its structure confirmed by NMR spectrometry. The second step (v) was also performed in an inert nitrogen atmosphere, in the dark at 0°C to RT. NMR spectrometry confirmed the formation of the phosphoramidite (**5**). Additional description of the synthesis of (*S*)-1-*O*-(4,4′-dimethoxytriphenylmethyloxy)-3-*O*-(2-(pyren-1-ylethynyl)benzyloxy)propan-2-ol (**4**) and (*S*)-1-*O*-(4,4′-dimethoxytriphenylmethyloxy)-3-*O*-(2-(pyren-1-ylethynyl)benzyloxy)-propan-2-yl 2-cyanoethyl diisopropylphosphoramidite (**5**) is in Supplementary data S1.

### Oligonucleotides and fluorescence resonance energy transfer (FRET) system

All oligonucleotides were purchased from IBA GmbH (Göttingen, Germany) or DNA Technology A/S (Risskov, Denmark) on a 0.2 µmol synthesis scale with high performance liquid chromatography (HPLC) purification and subsequently quality control.

Tm analysis targeted *E. coli rrs* gene base pairs 772–789 (5′-TGGGGAGCAAACAGGATT-3′). For ΔTm studies, mismatches were introduced at positions 3, 4, 6–12, 14, 16 and 18 from the 5′ position. Target oligonucleotides were synthesized with a 3′ amino-modifier-C7 and linked to ATTO495 NHS-ester (ATTO495 maximum excitation 495 nm; maximum emission 527 nm). The complementary sequence (5′-AATCCTGTTTGCTCCCCA-3′) was modified with one, two or three *ortho*- or *para*-TINA molecule insertions in the phosphate backbone at positions 0, 3, 6, 9, 12, 15 or 18 from the 5′ position, and 5′ labeled with ATTO590. ATTO590-modified oligonucleotides were synthesized with a 5′-amino-modifier-C6 and linked to an ATTO590 NHS-ester (ATTO590 maximum excitation 594 nm; maximum emission 624 nm). The ATTO495/ATTO590 FRET pair was excitated at 470 nm on a LightCycler®2.0 (Roche Applied Science, Basel, Switzerland); fluorescence emission was detected at 640 nm.

PCR product capture experiments used *E. coli rrs* gene base pairs 772–789 or 446–463, alongside equivalent sequences from *P. aeruginosa* as cross-reactivity control. Biotinylated PCR products were detected on the Luminex® 200™ instrument (Luminex Corp., Austin, TX, USA). Oligonucleotides were modified with an amino-modified cyclohexan (NH_2_-CX) and a hexaethylene glycol C18 spacer (HEG) to allow covalent coupling of the oligonucleotides to magnetic microspheres. *Ortho*- and *para*-TINA molecules were placed 5′ and 3′ terminally on the oligonucleotides. *E. coli rrs* gene sequence 772–789 was coupled to Luminex® MagPlex® microsphere number 61 as 5′-NH_2_-CX-HEG-AATCCTGTTTGCTCCCCA-3′. *E. coli rrs* gene sequence 446–463 was coupled to microsphere number 13 as 5′-ACTTTACTCCCTTCCTCC-HEG-CX-NH_2_-3′. The *P. aeruginosa* sequence – 5′-ACTTACTGCCCTTCCTCC-HEG-CX-NH_2_-3′ – was coupled to microsphere number 29. Helper oligonucleotide, targeting *E. coli rrs* gene base pair 464–483, was used without any modifications (5′-GTCAATGAGCAAAGGTATTA-3′).

### Melting curve acquisition

Melting curve experiments were performed on a LightCycler® 2.0 using 20 µL LightCycler® capillaries. 0.5 µM of each oligonucleotide was mixed with sodium phosphate buffer (50 mM NaH_2_PO_4_/Na_2_HPO_4_, 100 mM NaCl and 0.1 mM EDTA) at pH 7.0. Tm measurements were carried out using a standard program: (i) dissociation at 37 to 95°C, ramp rate 0.2°C/sec, 5 min hold at 95°C; (ii) annealing at 95 to 37°C, ramp rate 0.05°C/sec, continued measurement of fluorescence; (iii) 5 min hold at 37°C; and (iv) denaturation at 37 to 95°C, ramp rate 0.05°C/sec, and continued measurement of fluorescence. Tm was determined using fluorescence data from both the annealing and denaturation curves. No hysteresis was observed. Using LightCycler® Software 4.1 for melting curve analysis, Tm was defined as the peak of the first derivative. All melting curve determinations were conducted as single capillary measurements. A setup control (matching oligonucleotides D-624 and D-643) was included in all runs. Prior to Tm identification, runs were color compensated by subtraction of the fluorophore background fluorescence.

### Coupling of oligonucleotides to Luminex® MagPlex® microspheres

Conventional DNA oligonucleotides were coupled to MagPlex®-C magnetic carboxylated microspheres following the carbodiimide coupling procedure for amine-modified oligonucleotides, as recommended by Luminex Corporation. In short, 2.5×10^6^ microspheres were activated in 0.1 M MES, pH 4.5, followed by addition of 0.2 nmol oligonucleotide and 25 µg EDC. The coupling reaction was incubated for 30 min in the dark, followed by addition of 25 µg EDC and another 30 min incubation. 1.0 mL of 0.02% Tween-20 was added and the supernatant was removed after magnetic separation for 1 min on a DynaMag™-2 magnetic particle concentrator (Invitrogen A/S, Tåstrup, Denmark). 1 mL of 0.1% SDS was added and vortexed, followed by magnetic separation and resuspension in 100 µL Tris-EDTA buffer, pH 8.0, and refrigerated storage.

For *ortho*- and *para*-TINA modified oligonucleotides, a novel in-house carbodiimide/sulpho-NHS coupling procedure was followed. In a low retention microcentrifuge tube (Axygen, Union City, CA, USA), 2.5×10^6^ microspheres were washed and activated in 100 µL of 0.1 M MES, pH 6.0, then resuspended in 35 µL buffer. 125 µg sulpho-NHS was added, followed by 625 µg EDC, incubation in the dark for 15 min, addition of another 625 µg EDC and 15 min incubation. Activation buffer was removed and 97 µL of 0.1 M phosphate buffer, pH 7.2, was added followed by 0.3 nmol oligonucleotide. Microspheres were incubated for 2 hours at RT on a Thermo-shaker TS-100 (BioSan, Riga, Latvia) at 900 rpm, followed by optional overnight incubation, without shaking. Microspheres were washed once in 100 µL of 0.1 M phosphate buffer, pH 7.2, blocked in 0.1 M phosphate buffer with 50 mM ethanolamine, pH 7.2, and incubated for 15 min at RT on the Thermo-shaker at 900 rpm. Microspheres were separated and resuspended in 100 µL Tris-EDTA buffer, pH 8.0, and stored at 5°C. All separation steps involved placing the microcentrifuge tube in the magnetic separator for 1 min, with low speed vortexing for 20 sec after each addition of buffer or reagent.

To ensure equal coupling efficiency for the carbodiimide coupling procedure, and the carbodiimide/sulpho-NHS coupling procedure used for the *ortho*- and *para*-TINA modified oligonucleotides, a biotinylated oligonucleotide with or without terminally *para*-TINA modifications was included in each coupling protocol. The coupling efficiency was evaluated by incubation of 0.2 µL microspheres with 0.5 µg Streptavidin-R-PhycoErythrin Premium Grade (S-21388, Invitrogen A/S) with 10 µg albumin fraction V (Merck & Co Inc.), 0.03% Triton X-100 and 10 mM phosphate buffer, pH 6.4 with 200 mM NaCl. The reaction mixture was incubated for 15 min in an iEMS® Incubator/Shaker HT (Thermo Fisher Scientific) at 25°C and 900 rpm. After three washes in 10 mM phosphate buffer, pH 6.4, with 200 mM NaCl and 0.03% Triton X-100, 350 microspheres were counted on the Luminex® 200™ instrument. Similar coupling efficiencies were found using both procedures. Microspheres from a single coupling round were used in all experiments.

### *E. coli rrs* gene PCR with 40% biotin-11-dUTP and Luminex® 200™ assay detection

For the PCR product capture experiments, *E. coli* ATCC 25922 strain was harvested in exponential phase, and genomic DNA was purified using NucleoBond® AXG columns and Genomic DNA Isolation kit (Macherey-Nagel GmbH, Düren, Germany), following the manufacturer's protocol with addition of lysozyme as recommended. The concentration of genomic DNA was measured on a NanoDrop™ 1000 (Thermo Fisher Scientific Inc., Wilmington, USA) and diluted to 100 ng/µL.

PCR was performed in 25 µL reactions using 1x Euro Pink PCR Buffer (10.4 mM Tris-HCl, 56.8 mM Trizma-base, 16.1 mM (NH_4_)_2_SO_4_, 0.01% Tween80, 0.5% Ficoll400), 2 mM MgCl_2_, 0.08% BSA, 0.2 µM of each primer (FP-Eco-rrs-310: 5′-GCCACACTGGAACTGAGACA-3′ and RP-Eco-rrs-1033b: 5′-biotin-CCGAAGGCACATTCTCATCT-3′), 0.2 mM of each dNTP (0.08 mM dTTP was substituted by biotin-11-dUTP and 0.02 mM by dUTP), 1 U KAPA2G Robust HS and 100 ng genomic DNA in an Eppendorf® twin.tec 96-well PCR plate with Microseal® ‘B’ Film (BioRad Laboratories, Copenhagen, Denmark). PCR was performed on the CFX96™ Real-Time System (BioRad Laboratories). An endpoint PCR was performed: 3 min at 95°C; 35 cycles of denaturation at 95°C for 30 sec; annealing at 55°C for 25 sec; and extension at 72°C for 30 sec. The PCR amplicon was 723 base pairs long.

PCR products were pooled and purified using NucleoSpin® Extract II PCR clean-up (Macherey-Nagel GmbH). The purified product was evaluated by gel electrophoresis on a 1.5% agarose gel in TAE buffer with ethidium bromide staining with GeleRuler™ 100 bp Plus DNA Ladder (Fermentas GmbH, St. Leon-Rot, Germany). DNA concentration was 54.8 ng/µL, as determined by OD50 measurement on the NanoDrop™ 1000. The pooled PCR product was used in all experiments.

Biotinylated PCR products were detected on the Luminex® 200™ instrument (Luminex Corp.). A 70 µL premix of microspheres, PCR product, Triton X-100 and helper oligonucleotide (for *E.coli rrs* gene base pair 446–463 capture) was mixed in an Eppendorf® twin.tec 96-well PCR plate and incubated at 95°C for 10 min in a SensoQuest Labcycler (SensoQuest GmbH, Göttingen, Germany). The PCR plate was immediately transferred to ice for 2 min and 50 µL was transferred to a conical bottom 96 MicroWell™ Plate (NUNC, Thermo Fisher Scientific, Roskilde, Denmark) on ice, and 50 µL of a cold 2x hybridization buffer added. The final mixture consisted of 0.2 µL of the relevant microsphere (approximately 2,500 microspheres/well), a two-fold dilution series of biotinylated *E. coli rrs* gene PCR product from 2.5–0.0098 µL, 0.03% Triton X-100, and 1x hybridization buffer (20 mM NaH_2_PO_4_/Na_2_HPO_4_ adjusted with NaCl to monovalent cation concentrations of 100, 150, 200, 300, 400, 500 and 1000 mM at pH 7.0 (52°C)). The mixture was incubated for 15 min in an iEMS® Incubator/Shaker HT (Thermo Fisher Scientific) at 900 rpm and 52°C, or at 42, 46, 50, 54, 58 or 62°C in the temperature experiments. After incubation, the plate was washed three times by using a 96-well magnetic separator (PerkinElmer, Skovlunde, Denmark), removing the supernatant, and adding 20 mM NaH_2_PO_4_/Na_2_HPO_4_ adjusted with NaCl to 50 mM monovalent cation concentration and 0.03% Triton X-100 at pH 7.0. Next, 0.5 µg Streptavidin-R-PhycoErythrin Premium Grade (S-21388, Invitrogen A/S, Tåstrup, Denmark) with 10 µg albumin fraction V (Merck & Co Inc.), 0.03% Trition X-100 and 1x hybridization buffer, was added to each well. Plates were incubated for 15 min at 52°C (or relevant experimental temperature), and washed three times as previously described. Wash buffer was added, and incubated for 30 min at RT before Luminex® 200™ analysis, counting 300 of each microsphere set. The final step at RT avoided decreasing background fluorescence in the Luminex® analysis due to sedimentation of unevenly sized microspheres [23]. All dilution series were run in triplicate, with results presented as mean of MFI and 95% confidence intervals. Analytical sensitivity was defined as the limit of detection (LOD), calculated by adding three standard deviations to the mean background MFI. Differences in analytical sensitivity were defined as the ratio between the LOD of DNA and *ortho*- or *para*-TINA modified oligonucleotides.

## Supporting Information

Table S1 Change in Tm and ΔTm of Watson-Crick based antiparallel duplexes stabilized by *para* (X)- and/or *ortho* (Y)-TINA monomers. Tm was determined using 0.5 µM of each strand in 50 mM phosphate buffer, pH 7.0, with 100 mM NaCl and 0.1 mM EDTA. Tm was defined as the peak of the first derivative using both annealing and dissociation curves. Base mismatches are underlined and marked in bold blue. *Mismatch adjacent to TINA.
(XLS)

Figure S1Competitive annealing of *ortho*- or *para*-TINA terminally modified oligonucleotides compared with unmodified DNA oligonucleotide to denatured PCR products in buffer of increasing ionic strength – complete data. *E. coli rrs* biotinylated PCR product was captured by unmodified DNA oligonucleotide (▴) and *ortho*-TINA (•) or *para*-TINA (▪) modified oligonucleotides targeting *E. coli rrs* base pairs 772–789 and 446–463, with unmodified DNA oligonucleotide (▾), *ortho-*TINA (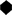) or *para*-TINA (⧫) modified oligonucleotides targeting *P. aeruginosa* base pairs 446–463 as cross-reactivity control. In experiments targeting base pairs 446–463, a conventional DNA helper oligonucleotide (base pair target 464–483) was included. Experiments were performed in phosphate buffer, pH 7.0, with 0.03% Triton X-100 and increasing ionic strength (100–1,000 mM) at 52°C. Data are presented as mean raw median fluorescence intensity (MFI) with 95% confidence intervals.(EPS)

Data S1 (DOC)
