# Supplementary material for: Increasing the Analytical Sensitivity by Oligonucleotides Modified with Para- and Ortho-Twisted Intercalating Nucleic Acids – TINA
Source: PLoS One. 2011 Jun 3;6(6):e20565. doi: 10.1371/journal.pone.0020565 (PMC3108614; doi:10.1371/journal.pone.0020565)
Supplement: Data S1 — (DOC) [file pone.0020565.s003.doc]

# Supplementary data S1 to ‘Materials and methods’

## (S)-1-O-(4,4′-dimethoxytriphenylmethyloxy)-3-O-(2-(pyren-1-ylethynyl)benzyloxy)propan-2-ol (compound 4)

Compound **3** (13) (3.04 g; 4.91 mmol) from Fig. 2 was dissolved in triethylamine (50 mL) and N_2_ was bubbled through the mixture for 10 min. CuI (0.072 g; 0.38 mmol) and Pd(Ph_3_)_3_Cl_2_ (0.098 g; 0.14 mmol) were added and the mixture was bubbled with N_2_ for another 5 min. 1-Ethynylpyrene (1.23 g; 5.45 mmol) was added and the reaction mixture was intensively bubbled with N_2_ for 5 min, followed by stirring at room temperature (RT) in the dark under an N_2_ atmosphere. The reaction was finished after 26 h according to silica TLC, and the reaction mixture was filtered through a bed of celite that was washed with CH_2_Cl_2_ (150 mL). The solvent was evaporated under reduced pressure and the crude product was purified by silica gel chromatography (cyclohexane:ethylacetate:triethylamine 100:100:1, v/v/v) producing compound **4** in 85% yield as a yellow foam (2.93 g; 4.13 mmol). ^1^H NMR (CDCl_3_) δ = 2.50 (d, *J* = 5.0 Hz, 1H, OH), 3.25 (m, 2H, CHOHC*H_2_*OCH_2_), 3.69 (s, 6H, 2 × OCH_3_), 3.77 (m, 2H, CH_2_ODMT), 4.00–4.16 (m, 1H, C*H*OH), 4.98 (s, 2H, CH_2_Ar), 6.71–6.75 (m, 4H, DMT), 7.17–7.29 (m, 8H, Ar), 7.36–7.45 (m, 4H, Ar), 7.48–7.50 (m, 1H, Ar), 7.66–7.80 (m, 1H, Ar), 7.94–8.30 (m, 8H, Ar), 8.65 (d, *J* = 9.1 Hz, 1H, Ar). ^13^C NMR (CDCl_3_) δ = 55.11 (2 × OCH_3_), 64.41 (CH_2_ODMT), 70.02 (CH(OH)*C*H_2_OCH_2_), 71.76 (OCH_2_-phenyl), 72.03 (CHOH), 86.08 (*C*Ph_3_), 92.72, 93.22 (C≡C), 113.06, 117.65, 122.28, 124.33, 124.51, 124.58, 125.45, 125.63, 125.72, 126.26, 126.70, 127.24, 127.61, 127.76, 128.01, 128.11, 128.24, 128.50, 128.59, 129.65, 130.02, 131.06, 131.25, 131.37, 131.87, 132.35, 135.99, 139.71, 144.84, 158.41 (Ar).

## (S)-1-O-(4,4′-dimethoxytriphenylmethyloxy)-3-O-(2-(pyren-1-ylethynyl)benzyloxy)-propan-2-yl 2-cyanoethyl diisopropylphosphoramidite (compound 5)

Compound **4** (0.5 g; 0.7 mmol) from Fig. 2 was dissolved in dry CH_2_Cl_2_ (5 mL) and N_2_ was bubbled through the mixture for 10 min. *N,N*-Diisopropylammonium tetrazolide (0.181 g; 1.06 mmol) was added and the mixture was bubbled with N_2_ for another 5 min. *N,N,N′,N′*-tetraisopropylphosphoramidite (0.67 mL; 2.11 mmol) was added drop wise to the mixture and stirred under an N_2_ atmosphere in the dark with external cooling from an ice bath. After 24 h, analytical TLC showed no more starting material (cyclohexane:ethylacetate:triethylamine 100:100:1, v/v/v) and the reaction mixture was quenched with H_2_O (25 mL) and extracted with CH_2_Cl_2_ (2 × 25 mL). The organic layers were combined, dried (MgSO_4_) and filtered through a bed of silica gel under reduced pressure (eluted with cyclohexane:ethylacetate:triethylamine 100:100:1, v/v/v) and evaporated under reduced pressure obtaining compound **5** in 85% yield as a yellow foam (0.545 g; 0.6 mmol).

^13^C NMR (CDCl_3_) δ = 20.18 (2 × *C*H_2_CN), 24.46, 24.51, 24.60, 24.67 (2 × CH(*C*H_3_)_2_), 43.17, 43.24 (2 × *C*H(CH_3_)_2_), 55.10 (2 × OCH_3_), 58.28, 58.30 (O*C*H_2_CH_2_CN), 64.45 (CHOP), 71.48 (CH*C*H_2_ODMT), 85.98 (*C*Ph_3_), 92.70, 93.21 (C≡C), 112.96, 117.73, 121.79, 124.31, 124.52, 124.56, 125.46, 125.62, 125.68, 126.27, 126.62, 127.23, 127.38, 127.67, 127.79, 128.15, 128.23, 128.46, 128.52, 129.61, 130.06, 130.11, 131.04, 131.24, 131.81, 132.09, 132.15, 136.15, 140.07, 144.93, 158.34 (Ar). ^31^P NMR (CDCl_3_) δ = 149.55, 149.37.
